# ENO2, a Glycolytic Enzyme, Contributes to Prostate Cancer Metastasis: A Systematic Review of Literature

**DOI:** 10.3390/cancers16142503

**Published:** 2024-07-10

**Authors:** Yuhan Zhou, Feier Zeng, Gareth Owain Richards, Ning Wang

**Affiliations:** 1 Division of Clinical Medicine, School of Medicine and Population Health, University of Sheffield, Sheffield S10 2RX, UK; 2 Leicester Cancer Research Centre, Department of Genetics and Genome Biology, University of Leicester, Leicester LE1 7LX, UK

**Keywords:** prostate cancer, ENO2, metastasis, androgen, glycolysis, neuroendocrine prostate cancer

## Abstract

**Simple Summary:**

This paper reviews the role of ENO2, a protein involved in sugar metabolism, in advanced prostate cancer. Analysing five studies, we found that ENO2 levels tend to be higher in aggressive forms of prostate cancer, particularly those that have spread or become resistant to hormone therapy. This increased presence might be linked to how prostate cancer cells change their energy production as the disease progresses, shifting to rely more on sugar breakdown in advanced stages. The study also suggests that ENO2 can be influenced by the tumour’s environment, such as low hormone levels or the presence of bone cells, which is relevant, as prostate cancer often spreads to bones. While not proving a direct causal relationship, the research indicates that ENO2 could be an important marker for aggressive disease and potentially a target for future treatments, warranting further investigation into its role in prostate cancer progression, especially in bone metastasis.

**Abstract:**

Prostate cancer (PCa) is the second leading cause of male cancer deaths in the UK and the fifth worldwide. The presence of distant PCa metastasis can reduce the 5-year survival rate from 100% to approximately 30%. Enolase 2 (ENO2), a crucial glycolytic enzyme in cancer metabolism, is associated with the metastasis of multiple cancers and is also used as a marker for neuroendocrine tumours. However, its role in PCa metastasis remains unclear. In this study, we systematically reviewed the current literature to determine the association between ENO2 and metastatic PCa. Medline, Web of Science, and PubMed were searched for eligible studies. The search yielded five studies assessing ENO2 expression in PCa patients or cell lines. The three human studies suggested that ENO2 expression is correlated with late-stage, aggressive PCa, including castrate-resistant PCa (CRPC), metastatic CRPC, and neuroendocrine PCa (NEPC). This was further supported by two in vitro studies indicating that ENO2 expression can be regulated by the tumour microenvironment, such as androgen deprived conditions and the presence of bone-forming osteoblasts. Therefore, ENO2 may functionally contribute to PCa metastasis, possibly due to the unique metabolic features of PCa, which are glycolysis dependent only at the advanced metastatic stage.

## 1. Introduction

PCa is the second most commonly diagnosed cancer in men, ranking as the fifth leading cause of cancer-related death in men worldwide [1] and second in the United Kingdom [2]. The 5-year survival rate for localised prostate cancer approaches 100%, whereas this rate markedly decreases to approximately 30% in cases exhibiting metastasis [3]. Unlike other cancer types, PCa has a unique metabolic feature, as primary PCa cells do not rely on the classic Warburg effect [4,5] until PCa reaches the metastatic advanced stage, when tumour cells increase their uptake of glucose and production of lactate through aerobic glycolysis, offering a more rapid ATP production rate to generate energy for survival and proliferation [6].

**Figure 1:**
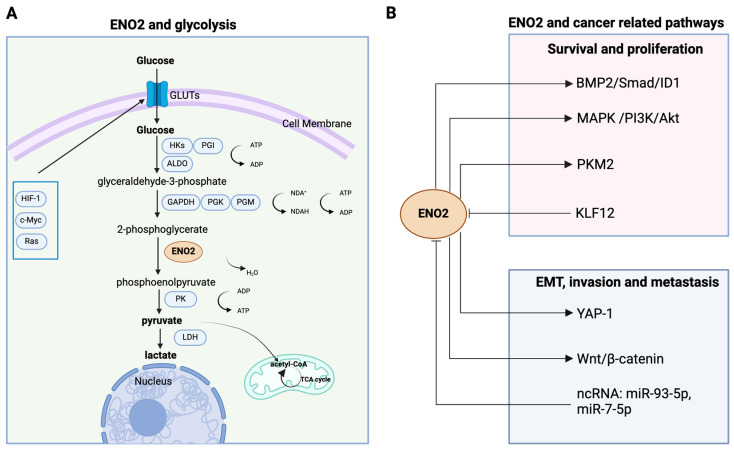
Schematic representation of the pleiotropic roles of ENO2 in cancer glycolysis and multiple pathways. (**A**) Enolase 2 catalyses the conversion of 2-phosphoglycerate into phosphoenolpyruvate and governs a critical step in the aerobic glycolytic pathway that converts glucose into pyruvate and ultimately into lactate. (**B**) In cancer cells, ENO2 plays diverse non-glycolytic roles, mediating cell proliferation, survival, EMT, invasion, and metastasis. ENO2 promotes cell proliferation and survival by activating PMK2, MAPK/PI3K/Akt, and BMP2/Smad/ID1 pathways. In addition, ENO2 augments tumour invasion and metastasis by regulating EMT via the YAP-1 and Wnt/β-catenin axis. Moreover, the activity of ENO2 can be affected by noncoding RNAs (e.g., miR-93-5p, miR-7-5p) and transcription factors such as KLF12. (Created with BioRender.com, agreement number: EH270PU3J9.).

ENO2, a crucial glycolytic metalloenzyme, participates in the penultimate step of glycolysis for synthesising pyruvate by catalysing the conversion of 2-phosphoglycerate (2PG) into phosphoenolpyruvate (PEP) in human cells [7]. Downstream signals activate pyruvate kinase (PK) and lactate dehydrogenase (LDH), promoting the conversion of PEP to pyruvate and ultimately to lactate, leading to a net gain of two molecules of ATP for each molecule of glucose [8]. Interestingly, tumour cells could enhance glycolysis by triggering pyruvate dehydrogenase kinase (PDK) activity, which in turn deactivates pyruvate dehydrogenase (PDH), obstructing the conversion of pyruvate into acetyl-CoA within the mitochondrial matrix (Figure 1A) [8]. It is notable that ENO2, involved in multiple signalling pathways, has been reported to be associated with the metastasis of multiple cancers, including but not limited to colorectal [9], pancreatic [10], and renal cancer [11], and is frequently linked to an unfavourable prognosis (Figure 1B). Furthermore, due to its predominant presence in the neurons and neuroendocrine cells, ENO2 is also widely used as a biomarker for the diagnosis and prognosis of poorly differentiated neuroendocrine tumours [7,12].

However, whether and how ENO2 contributes to PCa progression has not been elucidated. No studies have identified the connection between ENO2 and metastatic PCa development. We therefore undertook a systematic review to explore the association between ENO2 and PCa progression in order to understand the role of ENO2 in PCa malignant progression and uncover the possible underlying mechanism.

## 2. Materials and Methods

### 2.1. Protocol and Registration

The systematic review followed the recommendations of the Preferred Reporting Items for Systematic Reviews and Meta-Analyses (PRISMA). The protocol has not been registered.

### 2.2. Data Source and Searches

A protocol was conducted in accordance with the purpose-driven hypothesis. The Preferred Reporting Items for Systematic Reviews and Meta-Analyses (PRISMA) guidelines were utilised as a reference for this review. Keyword searches for “prostate cancer”, “prostate carcinoma”, “prostate neoplasm”, “ENO2”, “enolase 2”, “gamma enolase”, “neuron-specific enolase”, “nervous system-specific enolase”, “neuronal enriched enolase”, “2-phosphoglycerate dehydratase”, and “metastasis” were carried out using Web of Science, Medline, and PubMed. The developed search strategies for Web of Science, Medline, and PubMed are shown in Supplementary Table S1. Relevant articles were individually screened based on search strategy terms. Duplications were automatically removed using EndNote 20.6 (Clarivate Analytics, Philadelphia, PA, USA, 2023).

### 2.3. Inclusion and Exclusion Criteria

The selection criteria for this systematic review comprised both inclusion and exclusion criteria. Human studies that reported the association between ENO2 expression and PCa progression, as well as patient survival, were included. Studies that used in vitro models to test the association between ENO2 and PCa metastatic potential were also included. Reviews and case report articles were excluded. Studies that did not specifically address PCa and lacked clear data were excluded. Additionally, studies that were published in non-English languages were also excluded. 

### 2.4. Study Selection

After removing duplications, an initial screening process was employed, which involved screening the titles, abstracts, and keywords for a comprehensive list of keywords. Subsequently, the articles of the articles were screened secondarily based on the countries of origin, languages, type of articles, basic data, and research findings. Further screening of the full texts was carried out to identify eligible studies with figures containing relevant data. For the purpose of conducting the qualitative systematic review, a total of 5 articles were required. Two authors, Y.Z. and F.Z., independently conducted the abstract screening for eligibility. Any discordance was resolved through consensus with a third senior author, N.W.

### 2.5. Qualitiy Assessment

Risk of bias assessment of all eligible clinical and in vitro studies was assessed using the Newcastle–Ottawa Scale (NOS) and Office of Health Assessment and Translation (OHAT) risk of bias rating tool, respectively [13,14]. The following criteria were used for the NOS risk of bias rating: representativeness of the exposed cohort, selection of the non-exposed cohort, ascertainment of exposure, demonstration that the outcome of interest was not present at the start of the study, comparability of cohorts on the basis of the design or analysis, assessment of the outcome, follow-up long enough for outcomes to occur, and adequacy of the follow-up with the cohorts [13]. The following criteria were used for OHAT risk of bias rating: randomisation, allocation concealment, identical experimental conditions, blinding of researchers, complete outcome data, exposure characterisation, outcome assessment, outcome reporting, and other potential threats [14]. Studies with NOS scores of at least six were considered high quality, with higher scores indicating superior literature quality.

### 2.6. Certainty of Evidence

The certainty of evidence from eligible human and in vitro studies was evaluated using the Grading of Recommendations Assessment, Development, and Evaluation (GRADE) approach [15].

## 3. Results

### 3.1. Search Results

**Figure 2:**
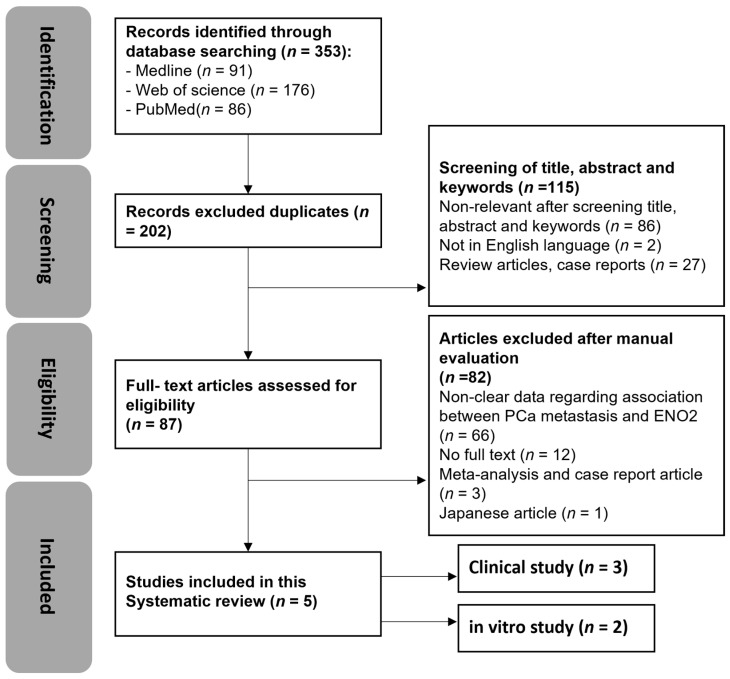
Systematic review flow diagram of evidence search and study selection process. *n* denotes the number of articles.

The search strategy employed in this study is detailed in Supplementary Table S1. An initial search was conducted on 12 May 2021, using Web of Science, Medline, and PubMed, and updated on 9 May 2024. A total of 353 articles were identified using the search strategy, including a total of 17 articles that had been updated in the past three years. As shown in Figure 2, 91 papers in Medline, 176 papers in Web of Science, and 86 papers in PubMed were identified, and 151 duplicates were removed. Out of the remaining 202 articles, 115 were excluded based on title, abstract, and keywords. A total of 87 articles were assessed for eligibility, 82 of which were excluded. The remaining included English-language articles (*n* = 5) that were categorised into human clinical and in vitro studies. No in vivo studies were identified during this search.

### 3.2. Quality Assessment 

All of the included clinical studies were scored as high quality and passed the quality assessment, as shown in Supplementary Table S2. Subsequently, all of the in vitro studies were scored as “probably high risk” for the criterion of “blinding of researchers” in Supplementary Table S3. However, this was of no concern to the authors since it is rather uncommon to blind researchers during in vitro experiments or specifically mention the blinding of in vitro experiments in publications [16]. Moreover, all of the in vitro studies were scored as “probably low risk” for the “complete outcome data” criterion. Out of the two included in vitro studies, one was considered “probably high risk” for the detection criterion (exposure characterisation and outcome assessment) and “definitely high risk” for the statistical methods criterion [17]. 

### 3.3. Certainty of Evidence

The outcomes of clinical studies that examined the ENO2 expression (mRNA and protein level by RNA-Seq, q RT-PCR, and serum biomarker analysis) in patients were uncertain due to non-randomised controlled trials and the small number of enrolled participants (very low certainty of evidence). Similarly, the in vitro mRNA level of ENO2 expression was uncertain (low certainty of evidence) due to the low study number.

### 3.4. Narrative Synthesis

#### 3.4.1. Clinical Studies

The included data from three clinical studies assessed the association between ENO2 expression, either in blood samples or downloaded from online datasets, and PCa malignant progression (Table 1).

Kim et al. performed a bioinformatic study that was carried out to validate their in vitro results by examining the ENO2 expression level through PCa gene expression profiling datasets using cBioPortal [18,19,20]. ENO2 tended to be strongly expressed in NEPC patients relative to both primary and CRPC patients. ENO2 expression was extremely upregulated in NEPC (*n* = 7) compared to primary PCa (*n* = 30) (*p* = 0.003) [19]. Concordantly, ENO2 expression was significantly increased in NEPC (*n* = 15) compared to CRPC (*n* = 34) (*p* = 0.002) [20]. Altogether, these data confirmed that ENO2 could be associated with NEPC. However, whether ENO2 contributes to the initiation and aggressive development of PCa cannot be concluded, as the original paper lacks a comparison between primary PCa and healthy controls, as well as between CRPC and primary PCa [18].

Kessel et al. conducted a cohort study to examine ENO2 expression by isolating circulating tumour cells (CTCs) from 19 patients with bone metastases (89%), lymph node metastases (68%), and visceral metastases (21%), with an average age of 68.8 years [21]. Ten of the blood samples from healthy donors were also collected to compare ENO2 expression levels with those of CRPC patients. ENO2 was dramatically upregulated in metastatic CRPC (mCRPC) patients compared with healthy individuals (*p* < 0.00005). The fact that all patients underwent distant metastases implies a connection between the malignant progression of PCa and high ENO2 expression. Furthermore, this study also found that ENO2 expression was significantly associated with mCRPC patients, both with and without a novel prognostic biomarker for mCRPC–androgen receptor (AR) splice variant 7 (AR-V7) [21]. However, the study has certain deficiencies due to the lack of comparison of ENO2 expression between patients with CRPC and primary PCa, as well as among healthy individuals and patients with primary PCa.

Szarvas et al. performed a cohort study to identify the serum ENO2 protein level using 1095 serum samples from 395 PCa patients [22]. A total of 157 hormone-sensitive patients who underwent radical prostatectomy (RP), 95 mCRPC patients treated with docetaxel (DOC) chemotherapy, and 143 mCRPC patients who underwent abiraterone/enzalutamide (ABI/ENZA) hormone therapy comprised the three cohorts in this study. Notably, the expression level of ENO2 in the mCRPC DOC group was significantly higher than that in the RP cohort (*p* = 0.001). Concordantly, the expression level of ENO2 in the mCRPC ABI/ENZA group was also significantly elevated compared to the RP group (*p* = 0.046). More importantly, ENO2 expression was significantly higher in patients with mCRPC than in hormone-naïve patients before treatment started. Taken together, this study indicated that the expression of ENO2 is upregulated in CRPC, especially the aggressive metastatic CRPC. This provided critical evidence by comparing ENO2 expression between primary PCa and CRPC, and filled the gap left by both Kim’s and Kessel’s research [22]. A univariate analysis in this paper also showed that the prognostic value of ENO2 is significant in ABI/ENZA-treated mCRPC patients, whereas it is not significant in DOC-treated mCRPC or PR-treated primary PCa patients, indicating the association between ENO2 level and PCa aggressiveness under the androgen-deprived condition.

**Table 1:** Characteristics of the included clinical studies of prostate cancer metastasis and ENO2.

Author	Year	Region	Study Type	No. of Patients	Mean Age (Years)	The Comparison Details	Comparison Outcome (*n* Number, *p* Value)
Kim et al. [18]	2017	USA	Bioinformatics (RNA-seq)	*n* = 86	/	The influence of ENO2 expression in patients with primary, CRPC, or NEPC in a clinical setting	NEPC (*n* = 15) > CRPC (*n* = 34) (*p* = 0.002) NEPC (*n* = 7) > Primary PCa (*n* = 30) (*p* = 0.003)
Kessel et al. [21]	2020	Germany	Cohort study (CTC enrichment and RT qPCR)	*n* = 19	68.8 years	The impact of ENO2 expression in blood samples obtained from either healthy individuals or mCRPC patients	mCRPC (*n* = 19) > Healthy controls (*n* = 10) (*p* < 0.00005)
Szarvas et al. [22]	2021	Germany	Cohort study (serum biomarker analysis)	*n* = 395	66 years (RP group) 71 years (DOC group) 73 years (ABI/ENZA group)	The effects of serum ENO2 protein level in patients who received radical prostatectomy or who received DOC or ABI/ENZA treatment in a clinical setting	mCRPC DOC > RP (*p* = 0.001) * mCRPC ABI/ENZA > RP (*p* = 0.046) * mCRPC 1st line pre-treatment > hormone-naïve pre-PRE (*p* > 0.001) *

CRPC: castration-resistant prostate cancer, NEPC: neuroendocrine prostate cancer, RP: radical prostatectomy, DOC: docetaxel, ABI: abiraterone, ENZA: enzalutamide. * *n* number not specified.

#### 3.4.2. In Vitro Studies

The two selected in vitro studies both examined ENO2 expression at transcriptional level by performing qRT-PCR assay using PCa cell lines (Table 2).

Bock et al. conducted a study using human osteoblast-derived microtissue models (hOBMT) co-cultured with distinct types of PCa cells to simulate the bone metastatic microenvironment [23]. In addition, PCa cells were cultured in two different mediums, either adequate or deficient in androgens in this model. The 10% FBS in normal RPMI medium was replaced with 10% charcoal-stripped serum (CSS) to simulate an androgen-deprived environment (PCa-AD). Subsequently, 10 nmol/L dihydrotestosterone (DHT) were applied to the PCa-AD medium as the PCa-DHT medium to simulate a living environment with sufficient androgens. Notably, ENO2 expression was relatively higher in the androgen-deficient environment compared to the adequate environment in all groups [23]. This is consistent with the aforementioned clinical studies showing that the expression of ENO2 is significantly upregulated in the androgen-deficient environment (CRPC) compared to the androgen-adequate environment (hormone-naive primary PCa or healthy individual). Intriguingly, regardless of whether in the PCa-AD or in the PCa-DHT medium, the relative expression of ENO2 in the osteotropic C4-2B cells co-cultured with hOBMT was upregulated compared to C4-2B cells cultured alone, while this was not observed in the non-bone tropic LNCaP cells, suggesting a possible association between ENO2 expression and the bone tropism of PCa cells. This is particularly important, as PCa predominantly metastasises to bone.

Bery et al. examined the expression levels of ENO2 in each of three different PCa cell lines: NCI-H660, 22Rv1, and PC3 [17]. The human PCa epithelial 22Rv1 cell line used in this study was derived from an androgen-dependent CWR22 xenograft and was collected from a patient with skeletal metastasis [24,25]. The human PCa epithelial androgen-independent PC3 cell line, which was also derived from bone metastasis, was also used in this study [26]. Additionally, NCI-H660, as the only epithelial neuroendocrine cancer cell line in this study, was identified as an extrapulmonary small cell carcinoma isolated from the prostate gland [27]. Notably, it was discovered that the expression level of ENO2 in 22Rv1, the only androgen-dependent cell line with bone metastasis potential, was higher than that in traditional neuroendocrine androgen-independent NCI-H660 cells [28]. Interestingly, the expression level of ENO2 in 22Rv1 cells, which are also PCa cells of non-neuroendocrine origin, was approximately three times higher than that in androgen-independent PC3 cells. Additionally, ENO2 expression in PC3 was the lowest among the three cell lines, approximately half that of NCI-H660 cells. However, owing to the absence of statistical analysis in the original article, whether ENO2 expression is associated with bone metastasis potential or AR status of the PCa cells is up for further investigation [17]. Despite the limited statistical power, this study shows all three PCa cell lines, no matter the metastatic or neuroendocrine status, exhibit detectable ENO2 expression. Additionally, ENO2 expression is not consistently higher in NEPC.

**Table 2:** Characteristics of the included in vitro studies of prostate cancer metastasis and ENO2.

Author	Year	Region	Assay Type	Cell Line	Culture Conditions	The Comparison Details	Comparison Outcome
Bock et al. [23]	2019	Australia	RT-qPCR	PC3	Monoculture medium: RPMI1640 + L-glutamine, 5%FBS + 1% P/S	The impact of ENO2 expression in vitro in PCa cell lines, which were either mono- or co-cultured with hOBMT in a medium with or without DHT	PCa-AD > PCa-DHT C4-2B (co-culture) > C4-2B (monoculture) LNCaP (mono- vs. co- culture): ns
				LNCaP	Co-culture medium: RPMI1640, 10%FBS (containing 0.6 nmol/L DHT) + 1%P/S (PCa-Norm) RPMI1640, 10%CSS + 1%P/S (PCa-AD)		
				C4-2B	RPMI1640, 10%FBS (containing 10 nmol/L DHT) + 1%P/S (PCa-DHT)		
Bery et al. [17]	2020	Germany	RT-qPCR	PC3	RPMI 1640, 5% FBS + 1% P/S	Expression levels of ENO2 in PCa cell lines of different types and origins	22Rv1 > NCI-H660 22Rv1 > PC3
				22Rv1	RPMI 1640, 10% FBS + 1% P/S		
				NCI-H660	RPMI1640, 0.005 mg/mL insulin + 0.01 mg/mL transferrin + 30 nM sodium selenite + 10 nM β-estradiol + 2 mM 5% FBS + 1% P/S		

hOBMT: human osteoblast-derived mineralised microtissue, DHT: dihydrotestosterone, CSS: charcoal-stripped serum, PCa-AD: androgen-deprived environment, PCa-DHT: androgen-adequate environment, monoculture: single cancer culture, co-culture: prostate cancer co-cultured with hOBMT, ns: not significant.

## 4. Discussion

Despite the growing armamentarium of treatments, including androgen deprivation, chemotherapy, receptor signalling inhibitors, and immunotherapies, metastatic PCa remains incurable. Metabolic reprogramming, as a hallmark of cancer, enables cancerous cells to meet increased nutrient and energy demands while withstanding the challenging microenvironment. Among the three enolase isoforms, both enolase 1 (ENO1) and ENO2 encode crucial glycolytic metalloenzymes and are frequently associated with metastases and unfavourable prognoses in multiple cancer types. ENO1 is the most extensively studied, with its role and mechanisms in cancer development having been thoroughly reviewed previously [7]. However, as ENO1 is widely expressed in normal cells and tissues, targeting ENO1 may impair normal cell functions and thereby increase the risk of side effects [7]. In contrast, with relatively limited expression in normal tissues, ENO2 presents a better target as a potential cancer therapy, especially for neuroendocrine tumours and particular cancer types [7,12].

Previous studies have shown that ENO2 is associated with metastasis and frequently an unfavourable prognosis in multiple cancers (Figure 1B). Recent studies revealed that BRAF V600E-mutated CRC cells displayed greater reliance on ENO2, thereby regulating proliferation, migration, and drug resistance in CRC cells by activating PI3K/Akt and MAPK pathways [29]. In addition, ENO2 has been found to interact with PKM2, preventing PKM2 ubiquitin-mediated proteasomal degradation and regulating PKM2-driven CCND1-mediated cell cycle progression, proliferation, and glycolysis in head and neck squamous cell carcinoma (HNSCC) [11]. Another study indicated that ENO2 triggered the activation of the BMP2/Smad/ID1 signalling pathway through its interaction with NBL1, thereby gaining stem cell-like properties in small cell lung cancer cells (SCLC) [30]. Further studies have examined the role of ENO2 in regulating multiple signalling pathways involved in tumour cell epithelial–mesenchymal transition (EMT). For instance, ENO2 has been shown to promote EMT through activating the Wnt/β-catenin pathway, thereby regulating tumour metastasis in SCLC [30]. Through knocking down and overexpressing ENO2, another study demonstrated ENO2′s role in driving the metastasis of colorectal cancer (CRC) cells by activating the YAP1-induced EMT process but was independent of glycolysis regulation [9]. Moreover, a recent study confirmed that silencing ENO2 notably reduced the expression levels of EMT markers, including N-cadherin and vimentin, in clear cell renal cell carcinoma (ccRCC) [31]. Interestingly, ENO2 has been identified to bind to kruppel-like factor 12 (KLF12) and repress its transcription at the promoter level. This interaction is negatively regulated by KLF12 and plays a tumour suppressor role in bladder cancer (BC) cells [32]. Notably, non-coding RNAs (ncRNAs) have been examined to associate with ENO2 in various ways to influence cell behaviours, particularly in the context of tumour metastasis and treatment resistance. For example, miR-7-5p and miR-93-5p were both reported to negatively regulate ENO2 and affect EMT and, subsequently, tumour invasion and metastasis in non-small cell lung cancer (NSCLC) [33] and in SCLC [34], respectively. Therefore, we focused this systematic review on investigating the association between ENO2 and prostate cancer metastasis. 

Evidence from the present systematic review indicates that ENO2 may become a promising target for elucidating the mechanisms underlying metastasis regulation, providing novel and timely insights. The systematic review yielded two key findings: (1) The expression of ENO2 is correlated with later, aggressive stages of PCa, including CRPC, mCRPC, and NEPC, suggesting ENO2 as a promising biomarker for advanced, hard-to-treat PCa; and (2) ENO2 expression can be altered by the tumour microenvironment, such as androgen deficiency and bone microenvironment, especially for osteotropic PCa cells. 

**Figure 3:**
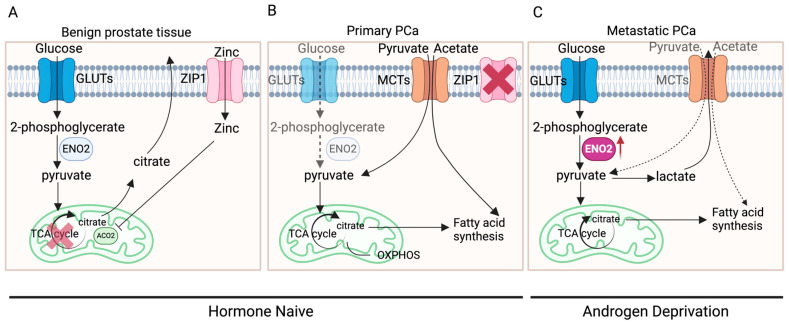
The metabolic switches in PCa development. (**A**) In benign prostate epithelial cells, zinc enters the cells through ZIP1 and acts as an inhibitor of ACO2, inhibiting the synthesis of citrate from pyruvate entering the TCA cycle. Citrate, synthesised using glucose, becomes an important component of prostatic fluid and the end product of anaerobic glycolysis. (**B**) In primary PCa, ZIP1 is significantly inhibited while pyruvate and acetate are obtained exogenously, leading to the entry of pyruvate into the TCA cycle, thereby exhibiting increased OXPHOS. (**C**) In metastatic PCa, ZIP1 expression is further inhibited, leading to markedly elevated aerobic glycolysis, which produces lactate. Part of the pyruvate enters the TCA cycle, generating citrate, which can be used as a substrate for fatty acid synthesis (created with BioRender.com, agreement number: SI270G6S6X).

The distinctive metabolic pattern of prostate cells might be a primary factor contributing to alterations in ENO2 expression. Normal prostate epithelial cells typically exhibit citrate-based metabolism, characterised by a relatively inefficient energy metabolic pattern using glucose and aspartic acid to synthesise citrate, an important component of prostatic fluid, and the final product of anaerobic glycolysis [6,35,36]. However, in the initiation and development of PCa, there are two metabolic switches: a conversion from anaerobic glycolysis to oxidative phosphorylation (OXPHOS) (benign prostate tissue to primary cancer) and a transition from OXPHOS to aerobic glycolysis and fatty acid synthesis (primary to advanced cancer) (Figure 3) [36]. During these switches, there are elements that have been identified to be key to the process, including AR, zinc transporter (ZIP1), and monocarboxylate transporters (MCTs).

ZIP1, a protein in humans encoded by *SLC39A1*, is responsible for the active transportation of zinc into prostate cells [37]. Within the cell, zinc acts as an inhibitor for aconitase 2 (ACO2), thereby suppressing the synthesis from pyruvate into citrate for entry into the tricarboxylic acid cycle (TCA cycle) to generate ATP [36,38]. In benign prostate tissue, when AR in the cytoplasm of prostate epithelial cells binds to its ligand DHT, the DHT-AR complex translocates to the nucleus and functions as a transcription factor for genes such as kallikrein-related peptidase *2 (KLK2*) and kallikrein-related peptidase *(KLK3)* [39]. This AR-mediated transcription stimulates prostate epithelial cells to produce citrate and regulates the expression of ZIP1 and glutamate–aspartate transporters (GLAST), thus promoting citrate synthesis [35]. During the oncogenic transformation in primary PCa tumours, efficient energy metabolism needs to be fulfilled for the proliferation of tumours instead of the production of citrate. AR-mediated metabolic reprogramming causes a metabolic shift towards OXPHOS and results in the inhibition of ZIP1 [35], leading to the entry of pyruvate into the TCA cycle and increased OXPHOS, a highly efficient process that produces more ATP compared to glycolysis [35,36,40]. More interestingly, PCa at this stage was shown to rely on the uptake of metabolic substrates other than glucose to fulfil their anabolic requirements [35,41,42]. Measurements of extracellular glucose uptake using a radiolabelled glucose analogue indicated that primary PCa exhibits minimal to no glucose uptake [43], whilst clinical imaging studies have demonstrated that PCa cells can absorb exogenous acetate and pyruvate that will be directly incorporated into the TCA cycle or enter the lipogenic metabolism [35,41,42]. In primary PCa cells, the uptake of exogenous pyruvate and acetate is achieved mainly through MCTs on the cell membrane, especially MCT2 [44]. 

Following PCa progression to later stages, especially metastasis, due to the requirement of excessive energy and nutrition under a challenging microenvironment, aerobic glycolysis is markedly elevated [35,36]. This could also be the consequence of hormone status alteration along with PCa progression. Most patients with primary PCa will need androgen deprivation therapy (ADT), while patients with CRPC may undergo second-generation hormone therapies, e.g., ABI and ENZI, to further inhibit the AR signalling by preventing androgen biosynthesis or AR translocation to the nucleus. These AR signalling-targeted therapies may remove the AR-mediated, OXPHOS-based metabolism and further favour aerobic glycolysis. Meanwhile, MCT4 expression is often increased in metastatic PCa to rapidly clear generated lactate and prevent intracellular acidification [45]. In contrast to OXPHOS, aerobic glycolysis adequately meets the energy demands of rapidly dividing tumour cells [40,46]. The elevated glucose uptake serves as the anabolic carbon source required for malignant proliferation of tumour cells [4]. Tumour cells require not only energy but also metabolic intermediates for biosynthesising macromolecules crucial for their growth and propagation. Numerous intermediates are generated by glycolysis that can be used for the biosynthesis of essential macromolecules, including lipids, nucleic acids, and proteins, supporting the accelerated proliferation of cancer cells [40,47]. At the later stage of PCa development and progression, due to the switch in metabolism, the expression of ENO2 is enhanced as it is functionally embedded within the glycolytic process. 

Taken together, considering this unique metabolic transition in metastatic PCa, targeting ENO2 offers promising potential in the treatment of advanced, hard-to-treat PCa. Therefore, further investigation and functional verification of ENO2′s role in AR-mediated PCa metabolic switch and progression of PCa are warranted.

## 5. Conclusions

In summary, the present systematic review demonstrates that ENO2 expression is associated with PCa progression, especially metastasis. The functional contribution is possibly due to the unique metabolic features of PCa, which are glycolysis dependent at the advanced metastatic stages. However, there are no specific functional studies that have identified the underlying molecular mechanism. Therefore, further validation and investigation of targeting ENO2 in advanced PCa development, especially in regulating bone metastasis, is innovative and clinically needed.

## Data Availability

The data presented in this study are available in this article and Supplementary Materials.

## Supplementary Materials

The following supporting information can be downloaded at: https://www.mdpi.com/article/10.3390/cancers16142503/s1, Table S1: List of search strategies of Web of Science, Ovid MEDLINE^®^ and PubMed; Table S2: Quality assessment for clinical studies using a modified Newcastle-Ottawa scale (NOS); Table S3: Quality assessment for in vitro studies using the OHAT RoB tool.

## Abbreviations

2PG	2-Phosphoglycerate
ABI/ENZA	Abiraterone/enzalutamide
ACO2	Aconitase 2
ADT	Androgen deprivation therapy
AR	Androgen receptor
BC	Bladder cancer
BMP2	Bone morphogenetic protein 2
BRAF	B-Raf proto-oncogene, serine/threonine kinase
CCND1	Cyclin D1
CRC	Colorectal cancer
CRPC	Castrate-resistant PCa
CSS	Charcoal-stripped serum
CTCs	Circulating tumour cells
DHT	Dihydrotestosterone
DOC	Docetaxel
EMT	Epithelial mesenchymal transition
ENO2	Enolase 2, Γ-enolase
GLAST	Glutamate–aspartate transporter
GRADE	Grading of Recommendations Assessment, Development, and Evaluation
HNSCC	Head and neck squamous cell carcinoma
hOBMT	Human osteoblast-derived microtissues
ID1	Inhibitor of DNA binding 1
KLF12	Kruppel-like factor 12
KLK2	Kallikrein-related peptidase 2
KLK3	Kallikrein-related peptidase 3
LDH	Lactate dehydrogenase
ncRNAs	Non-coding RNAs
MAPK	Mitogen-activated protein kinase
mCRPC	Metastatic CRPC
MCT	Monocarboxylate transporters
NBL1	DAN family BMP antagonist
NEPC	Neuroendocrine prostate cancer
NEtD	Transdifferentiation
OXPHOS	Oxidative phosphorylation
PCa	Prostate cancer
PDH	Pyruvate dehydrogenase
PDK	Pyruvate dehydrogenase kinase
PEP	Phosphoenolpyruvate
PI3K	Phosphatidylinositol 3-kinase
PK	Pyruvate kinase
PRISMA	Reporting Items for Systematic Reviews and Meta-Analyses
RP	Radical prostatectomy
SCLC	Small cell lung cancer cells
SLC39A1	Solute carrier family 39 member 1
TCA cycle	Tricarboxylic acid cycle, citric acid cycle
YAP1	Yes1-associated transcriptional regulator
ZIP1	Zinc transporter
