# Supplementary material for: ENO2, a Glycolytic Enzyme, Contributes to Prostate Cancer Metastasis: A Systematic Review of Literature
Source: Cancers (Basel). 2024 Jul 10;16(14):2503. doi: 10.3390/cancers16142503 (PMC11274830; doi:10.3390/cancers16142503)
Supplement: Supplementary file 1 [file cancers-16-02503-s001.zip › Table S1.pdf]

Supplementary Table S1. List of search strategies of Web of Science, Ovid MEDLINE® and PubMed.

Web of Science	Ovid MEDLINE®	PubMed
TS= ('cancer' OR '*carcinoma' OR 'tumo$r' OR 'neoplasm$')	(cancer or carcinoma or tumo$r or neoplasm$).mp.	((("prostate cancer"[All Fields]) OR ("prostate carcinoma"[All Fields])) OR ("prostate neoplasms"[All Fields])) OR ("prostate tumour"[All Fields])
TS= ('ENO2 protein$' OR 'ENO2 Gene' OR 'ENO2)	(ENO2 protein$ or ENO2 Gene or ENO2).mp.	(((("cancer"[All Fields]) OR ("tumor"[All Fields])) OR ("tumour"[All Fields])) OR ("carcinoma"[All Fields])) OR ("neoplasma"[All Fields])
TS= ('Enolase 2')	Enolase 2.mp.	(("eno2"[All Fields]) OR ("eno2 protein"[All Fields])) OR ("eno2 gene"[All Fields])
TS= ('Gamma Enolase' OR Gamma-Enolase')	(Gamma Enolase or Gamma- Enolase).mp.	("enolase 2"[All Fields]) OR ("gamma enolase"[All Fields])
TS= ('Neuronal Enriched Enolase' OR 'Neural Enolase')	(Neuronal Enriched Enolase or Neural Enolase).mp.	Neuronal Enolase
TS= ('Neuron-Specific Enolase' OR 'Neuron Specific Gamma Enolase' OR 'Neuron-Specific Gamma Enolase' OR 'NSE')	(Neuron-Specific Enolase or Neuron Specific Gamma Enolase or Neuron- Specific Gamma Enolase or NSE).mp.	(("neuron specific gamma enolase"[All Fields]) OR ("neuron specific enolase"[All Fields])) OR ("nse"[All Fields])
TS= ('2-Phospho-D-Glycerate Hydro$Lyase' OR '2 Phospho D Glycerate Hydro$Lyase')	2-Phospho-D-Glycerate Hydro$Lyase or 2 Phospho D Glycerate Hydro$Lyase).mp.	"2 phospho d glycerate hydro lyase"[All Fields]
TS= ('2-Phosphoglycerate Dehydratase' OR '2 Phosphoglycerate Dehydratase')	(2-Phosphoglycerate Dehydratase or 2 Phosphoglycerate Dehydratase).mp.	"2 phosphoglycerate dehydratase"[All Fields]
TS= ('Nervous System-Specific Enolase' OR 'Nervous System Specific Enolase')	(Nervous System-Specific Enolase or Nervous System Specific Enolase).mp.	"nervous system specific enolase"[All Fields]

TS= (‘prostat* Carcinoma$’ OR ‘carcinom$, prostate’)	(prostat* Carcinoma$ or carcinom$, prostate).mp.	((("metastasis"[All Fields]) OR ("metastatic"[All Fields])) OR ("metastases"[All Fields])) OR ("metastasizes"[All Fields])
TS= (‘prostat* Cancer’ OR ‘cancer, prostate’)	(prostat* Cancer or cancer, prostate).mp.	#3 OR #4 OR #5 OR #6 OR #7 OR #8 OR #9
TS= (‘prostat* tumo$r’ OR ‘tumo$r, prostate’)	prostat* tumo$r or tumo$r, prostate).mp	#1 AND #2 AND #10 AND #11
TS= (‘prostat* neoplasm$’ OR ‘neoplasm$, prostate’)	(prostat* neoplasm$ or neoplasm$, prostate).mp.	#1 AND #2 AND #10 AND #11 Filters: Review
TS= (‘metastas*s’)	metastas*s.mp.	#12 NOT #13
#2 OR #3 OR #4 OR #5 OR #6 OR #7 OR #8 OR #9	2 or 3 or 4 or 5 or 6 or 7 or 8 or 9	
#10 OR #11 OR #12 OR #13	10 or 11 or 12 or 13	
#1 AND #14 AND #15 AND #16	1 and 14 and 15 and 16	
#1 AND #14 AND #15 AND #16 and Preprint Citation Index (Exclude – Database) and Review Article (Exclude – Document Types)	limit 17 to "review articles"<br>17 not 18	
