# Supplementary material for: ENO2, a Glycolytic Enzyme, Contributes to Prostate Cancer Metastasis: A Systematic Review of Literature
Source: Cancers (Basel). 2024 Jul 10;16(14):2503. doi: 10.3390/cancers16142503 (PMC11274830; doi:10.3390/cancers16142503)
Supplement: Supplementary file 1 [file cancers-16-02503-s001.zip › Table S2.pdf]

Supplementary Table S2. Quality assessment for clinical studies using a modified Newcastle-Ottawa scale (NOS).

**Study ID**	**Selection**				**Comparability**	**Outcomes**			**Total (out of 10)**
	Representativeness of the exposed cohort (☆☆)	Selection of the non-exposed cohort (☆)	Ascertainment of exposure (☆☆)	Demonstration that outcome of interest was not present at start of study (☆)	Comparability of cohorts on the basis of the design or analysis (☆☆)	Assessment of outcome (☆☆)	Was follow-up long enough for outcomes to occur (☆)	Adequacy of follow up of cohorts (☆☆)	
Kim et al. 2017	☆	☆	☆	☆	☆	☆	☆	☆	☆☆☆☆☆☆ (6)
Kessel et al. 2020	☆	/	☆	☆	☆	☆	☆	☆	☆☆☆☆☆☆☆ (7)
Szarvas et al. 2021	☆	☆	☆	☆	☆	☆	☆	☆	☆☆☆☆☆☆☆☆ (8)

Good quality: 3 or 4 ☆ in selection domain and 1 or 2 ☆ in comparability domain and 2 or 3 ☆ in outcome/exposure domain.

Fair quality: 2 ☆ in selection domain and 1 or 2 ☆ in comparability domain and 2 or 3 ☆ in outcome/exposure domain.

Poor quality: 0 or 1 ☆ in selection domain or 0 ☆ in comparability domain or 0 or 1 ☆ in outcome/exposure domain.
