# Supplementary material for: ENO2, a Glycolytic Enzyme, Contributes to Prostate Cancer Metastasis: A Systematic Review of Literature
Source: Cancers (Basel). 2024 Jul 10;16(14):2503. doi: 10.3390/cancers16142503 (PMC11274830; doi:10.3390/cancers16142503)
Supplement: Supplementary file 1 [file cancers-16-02503-s001.zip › Table S3.pdf]

Supplementary Table S3. Quality assessment for in vitro studies using the OHAT RoB tool.

Bias domain	Questions	Study ID	
		Bock et al. 2019	Bery et al. 2020
**Selection**	*randomization*	++	++
	allocation concealment	++	++
**Performance**	*identical experimental conditions*	++	++
	blinding of researchers	--	--
**Exclusion**	*complete outcome data*	+	+
**Detection**	*exposure characterization*	++	-
	outcome assessment	++	-
**Selective Reporting**	*outcome reporting*	++	++
**Other potential threats**	*statistical methods*	++	--

'++' designate definitely low risk of bias, '+' designate probably low risk of bias, '-'designate probably high risk of bias, and '--' designate definitely *high risk of bias.*
